# Interoperable Electronic Health Records and Health Information Exchanges: Systematic Review

**DOI:** 10.2196/12607

**Published:** 2019-06-06

**Authors:** Mark J Dobrow, Jessica P Bytautas, Sukirtha Tharmalingam, Simon Hagens

**Affiliations:** 1 Institute of Health Policy, Management and Evaluation Dalla Lana School of Public Health University of Toronto Toronto, ON Canada; 2 Canada Health Infoway - Inforoute Santé du Canada Toronto, ON Canada

**Keywords:** health information exchange, electronic health record, interoperability, use, impact, systematic review

## Abstract

**Background:**

As the availability of interoperable electronic health records (iEHRs) or health information exchanges (HIEs) continues to increase, there is greater need and opportunity to assess the current evidence base on what works and what does not regarding the adoption, use, and impact of iEHRs.

**Objective:**

The purpose of this project is to assess the international evidence base on the adoption, use, and impact of iEHRs.

**Methods:**

We conducted a systematic review, searching multiple databases—MEDLINE, Embase, and the Cumulative Index to Nursing and Allied Health Literature (CINAHL)—with supplemental searches conducted in Google Scholar and grey literature sources (ie, Google, Grey Literature Report, and OpenGrey). All searches were conducted in January and February 2017. Articles were eligible for inclusion if they were published in English, were published from 2006 to 2017, and were either an original research study or a literature review. In order to be included, articles needed to focus on iEHRs and HIEs across multiple health care settings, as well as on the impact and effectiveness of iEHR adoption and use.

**Results:**

We included 130 articles in the synthesis (113 primary studies, 86.9%; 17 reviews, 13.1%), with the majority focused on the United States (88/130, 67.7%). The primary studies focused on a wide range of health care settings; the three most prevalent settings studied included acute care (59/113, 52.2%), primary care (44/113, 38.9%), and emergency departments (34/113, 30.1%). We identified 29 distinct measurement items in the 113 primary studies that were linked to 522 specific measurement outcomes. Productivity and quality were the two evaluation dimensions that received the most attention, accounting for 14 of 29 (48%) measurement items and 306 of 522 (58.6%) measurement outcomes identified. Overall, the majority of the 522 measurement outcomes were positive (298/522, 57.1%). We also identified 17 reviews on iEHR use and impact, 6 (35%) that focused on barriers and facilitators to adoption and implementation and 11 (65%) that focused on benefits and impacts, with the more recent reviews finding little generalizable evidence of benefit and impact.

**Conclusions:**

This review captures the status of an evolving and active field focused on the use and impact of iEHRs. While the overall findings suggest many positive impacts, the quality of the primary studies were not evaluated systematically. When broken down by specific measurement item, the results directed attention both to measurement outcomes that were consistently positive and others that were mostly negative or equivocal.

## Introduction

Interoperable electronic health records (iEHRs) in Canada increasingly provide individual patients with a secure and private record of their health history and care within their health system [1]. The iEHR draws on core systems that collect information electronically, including client and provider demographic registries, diagnostic imaging systems, drug information systems, laboratory information systems, public health systems, and clinical reporting systems [2]. This record is designed to facilitate the sharing of data across the continuum of care, across health care delivery organizations, and across geographical areas. In Canada, 42% of nurses and 42% of primary care physicians report having access to provincial and territorial patient information systems [3,4]. However, the method to access information, the availability of information in care settings, and the user information needed to access information differs across provinces and territories.

While different in architecture, iEHR solutions are analogous to health information exchange (HIE) initiatives in the United States. Health professionals in Canada, as in most high-income countries, also receive patient information across settings through other digital health solutions, such as hospital information systems and laboratory systems. Other countries have developed systems similar to Canada’s iEHRs. The common element of interest for this project is the provision of information across care settings and health professionals to improve care for patients.

As the availability of iEHRs continues to increase [2], there is greater need and opportunity to assess and understand the current evidence base on what works and what does not work regarding the adoption, use, and impact of iEHRs. This evidence is important to guide progress and improve iEHR capabilities but also to identify gaps in the evidence base where more targeted investment and evaluation is needed. Therefore, the purpose of this project is to conduct a systematic review of the international evidence base on the adoption, use, and impact of iEHRs or HIEs. The findings will also contribute to a national study to value the contribution of iEHRs and other connected information, which is part of a series of studies to value key digital health benefits [5].

## Methods

We conducted a systematic review, documenting the key elements of the review, including search strategy, eligibility criteria, article selection process, analysis, and synthesis, using the Preferred Reporting Items for Systematic Reviews and Meta-Analyses (PRISMA) checklist.

### Search Strategy

We developed and tested multiple search strategies that incorporated subject heading and keyword terms for “interoperable electronic health record” (including “iEHR”), “health information exchange” or “HIE”; “interoperability”; “adoption” or “use”; and “effectiveness,” “impact,” or “value.” We consulted and sought feedback on the search strategies with health informatics experts, including from academic and government agencies each focused on health informatics, and a specialist librarian who performed an abbreviated Peer Review of Electronic Search Strategies (PRESS) assessment of a preliminary MEDLINE search strategy [6]. The health informatics experts identified five articles that reflected the targeted aims of the review, which we used to test the effectiveness of candidate search strategies to identify relevant articles. Ultimately, a final search strategy was selected and translated for use in several traditional databases—MEDLINE, Embase, and the Cumulative Index to Nursing and Allied Health Literature (CINAHL)—with supplemental searches conducted in Google Scholar and grey literature sources (ie, Google, Grey Literature Report, and OpenGrey); see Multimedia Appendix 1 for database-specific search strategies. All searches were conducted in January and February 2017. Additionally, reference lists of all included articles were reviewed to identify additional articles that the search strategy missed.

### Eligibility Criteria

Articles were eligible for inclusion in the review if they were published in English; published between 2006 and 2017, although, only a small number of articles published in 2017 were available at the time of our review; and were either an original research study, inclusive of both quantitative and qualitative study types, or a literature review. For inclusion in the review, articles needed to focus on iEHRs and HIEs, excluding electronic medical records, across two or more health care settings; they also needed to focus on the impact or effectiveness of iEHR or HIE adoption or use.

### Study Selection

Two reviewers (JPB and one of three research assistants) independently screened all titles and abstracts or, in the case of Google and Google Scholar search results, titles and excerpts. Discrepancies were resolved through discussion and/or review by a third reviewer (MJD). The same process was used for review of full-text articles.

### Analysis and Synthesis

Data from all articles identified for inclusion were extracted independently by two reviewers (JPB and one of three research assistants) using a predeveloped data extraction template that included the year of publication, article type, approach, methodology, jurisdiction, and health care setting. Given the anticipated heterogeneity of study types and the intention to capture the breadth of iEHR evaluation activity, we prioritized evaluation relevance over quality and, therefore, did not use available quality criteria to exclude primary studies [7-9].

**Figure 1:**
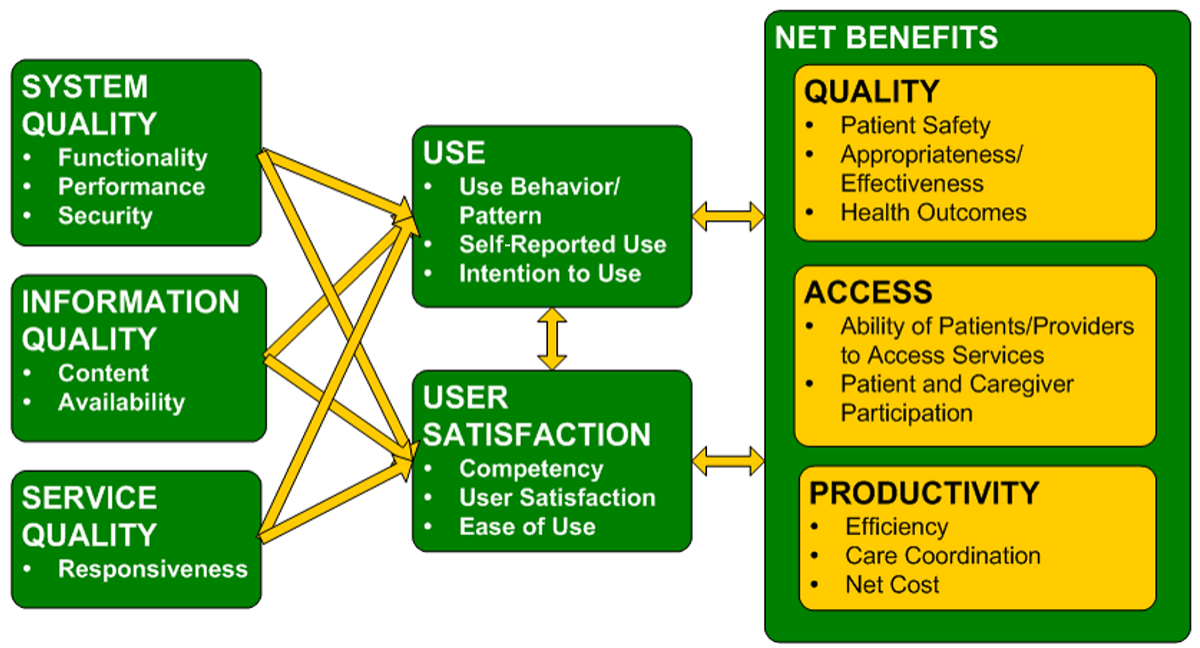
Canada Health Infoway benefits evaluation framework.

For all primary studies, two reviewers (JPB and one of three research assistants) independently extracted distinct measurement items and measurement outcomes verbatim. Measurement items were then coded and recoded inductively over three iterations into thematic categories by one reviewer (JPB) in discussion with the review team. All measurement outcomes were categorized as positive, negative, or mixed or neutral by one reviewer (JPB) and reviewed by the review team. All thematic measurement item categories were classified as one of the eight evaluation dimensions of benefit—system quality, service quality, information quality, user satisfaction, use, productivity, quality, and access—based on the Infoway benefits evaluation framework (see Figure 1). This framework, based on the Delone and MacLean Information Systems Success Model [10], details the measurement item and outcome categories and has been used extensively across Canada and internationally since it was first published in 2007 [11]. This classification approach has also been used in a relevant recent review of project evaluations from electronic health record (EHR) implementations across Canada [12].

We employed a separate analysis approach for the included literature review articles, conducting a descriptive analysis of each review article that assessed the (1) main focus of the review, (2) main findings, and (3) recommendations for future research.

## Results

### Overview

**Figure 2:**
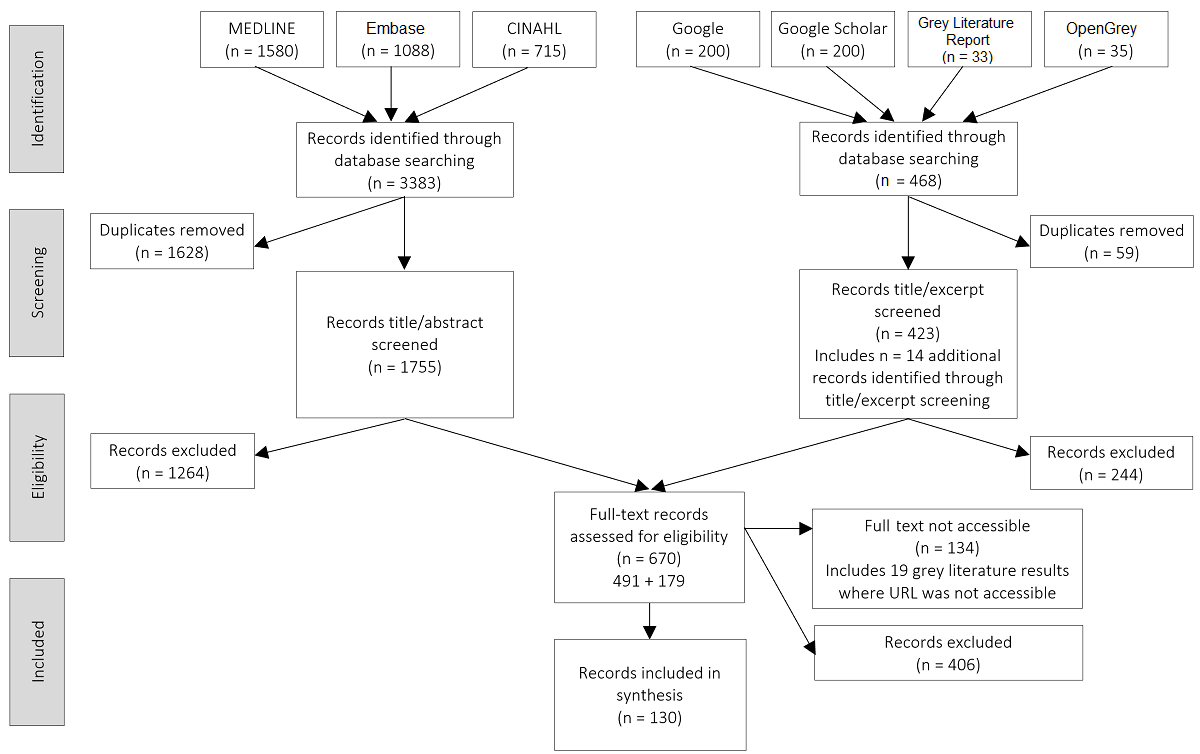
Preferred Reporting Items for Systematic Reviews and Meta-Analyses (PRISMA) flow diagram. CINAHL: Cumulative Index to Nursing and Allied Health Literature.

Our search of seven data sources identified 3851 records. After deduplication; title, abstract, and excerpt screening; and full-text review, 130 articles were included in the synthesis; Figure 2 presents the PRISMA flow diagram for the review. These 130 articles included 113 journal articles (86.9%), 11 reports (8.5%), and 6 documents of other types (4.6%). Of the 130 articles, 113 were primary studies (86.9%) and 17 were various types of reviews (13.1%). The vast majority of the articles focused on the United States (88/130, 67.7%); 6 (4.6%) focused on Israel; 3 (2.3%) each focused on Canada, Finland, and the United Kingdom; 2 (1.5%) focused on South Korea; 7 (5.4%) focused on another single country, including Australia, Austria, Brazil, Greece, Kenya, the Netherlands, and Switzerland; and another 18 (13.8%) had a multi-country focus. Of the 130 articles, 95 (73.1%) were published in the 6-year period from 2012 to 2017.

For the 113 primary studies, the majority employed quantitative methodologies exclusively (71/113, 62.8%) or in combination with qualitative methods (ie, mixed-method approaches) (20/113, 17.7%). The primary studies focused on a wide range of health care settings; the three most prevalent settings studied included acute care (59/113, 52.2%), primary care (44/113, 38.9%), and emergency departments (34/113, 30.1%). Other settings included laboratories (18/113, 15.9%), ambulatory care (17/113, 15.0%), pharmacies (13/113, 11.5%), public health departments (14/113, 12.4%), long-term care (9/113, 8.0%), payer or purchaser organizations (9/113, 8.0%), and home and community care (3/113, 2.7%).

### Summary of Findings From Primary Studies

The primary studies focused on six general dimensions. We identified 29 distinct measurement items, representing evaluation themes, in the 113 primary studies that were linked to 522 specific measurement outcomes (see Table 1). Productivity and quality were the two evaluation dimensions that received the most attention in the articles we reviewed, accounting for 14 of 29 (48%) measurement items identified and 306 of 522 (58.6%) measurement outcomes identified. Of the six evaluation dimensions assessed, service quality and system quality received the least attention, accounting for only 5 of 29 (17%) measurement items and 79 of 522 (15.1%) measurement outcomes documented from the articles reviewed. Measurement items were not assigned to either the *use* or *access* evaluation dimensions.

Overall, the majority of the 522 measurement outcomes were positive (298/522, 57.1%), with the remaining measurement outcomes reported as neutral or mixed results (107/522, 20.5%) or negative findings (117/522, 22.4%). When examining each of the 29 measurement items separately, the majority (22/29, 76%) had more positive than negative measurement outcomes, with the most frequently studied measurement items having a larger proportion of positive over negative outcomes. The 5 measurement items (5/29, 17%) with more negative than positive measurement outcomes were (1) stakeholder engagement, (2) performance and reliability, (3) security and privacy, (4) overall quality of information, and (5) ease of use; 2 measurement items (2/29, 7%) had equal positive and negative measurement outcomes: (1) layout and format and (2) community-based care, public or population health, or preventive services.

To provide more details on the findings, we consider each of the six dimensions separately. A total of 2 measurement items (2/29, 7%) were aligned with the service quality dimension. These included stakeholder engagement, which has mostly negative results, and training and support, which has mostly positive results. For the system quality dimension, 3 of 29 (10%) measurement items applied, including performance and reliability, security and privacy, and assessment and planning. Of these 3, only the latter had positive measurement outcomes. The information quality dimension had 6 of 29 (21%) measurement items, with the 2 most frequently measured items—data accuracy and completeness, as well as information availability—mostly positive, while the 4 less frequently measured items each revealed equivocal results. A total of 4 of 29 measurement items (14%) aligned with the user satisfaction dimension, with 1 showing mostly positive measurement outcomes—perceived usefulness or value and trust or confidence in the system—while the remaining 3 measurement items showed measurement outcomes that were either equivocal or negative. The productivity and quality dimensions each had 7 of 29 (24%) measurement items. All 7 measurement items for productivity had positive measurement outcomes, while 6 of the 7 measurement items for quality also had positive measurement outcomes. As noted above, the productivity and quality dimensions have received the majority of focus for measurement and have yielded mostly positive outcomes.

**Table 1:** Classification of iEHR^a^ and HIE^b^ measurement outcomes from primary studies. Measurement items are ordered by dimension and then by total number of measurement outcomes. Identified measurement items are only reported once in the table.

Evaluation dimension, measurement item		N	Positive, n (%)	Mixed or neutral, n (%)	Negative, n (%)	Total, n (%)
**Productivity**					
	Financial costs	58	28 (48)	16 (28)	14 (24)	58 (100)
	Efficiency in ordering and accessing tests, exams, results, or other clinical info	53	37 (70)	14 (26)	2 (4)	53 (100)
	Time savings in general	19	16 (84)	0 (0)	3 (16)	19 (100)
	Reduced hospital admissions and readmissions; shorter length of stay	19	10 (53)	5 (26)	4 (21)	19 (100)
	General productivity	13	10 (77)	2 (15)	1 (8)	13 (100)
	Efficiency due to improved organizational and managerial effectiveness	6	6 (100)	0 (0)	0 (0)	6 (100)
	Return on investment	2	2 (100)	0 (0)	0 (0)	2 (100)
	Subtotal	170	109 (64)	37 (22)	24 (14)	170 (100)
**Quality**					
	Enhanced ability to communicate, collaborate, and coordinate care	41	29 (71)	8 (20)	4 (10)	41 (100)
	Overall quality of care	26	18 (69)	6 (23)	2 (8)	26 (100)
	Clinical decision support	24	18 (75)	3 (13)	3 (13)	24 (100)
	Prescribing behavior, medication monitoring, or support	19	9 (47)	8 (42)	2 (11)	19 (100)
	Patient health outcomes	9	6 (67)	3 (33)	0 (0)	9 (100)
	Patient safety	9	5 (56)	4 (44)	0 (0)	9 (100)
	Community-based care, public or population health, or preventive services	8	3 (38)	2 (25)	3 (38)	8 (100)
	Subtotal	136	88 (65)	34 (25)	14 (10)	136 (100)
**Information quality**					
	Accuracy and completeness of data	22	12 (55)	5 (23)	5 (23)	22 (100)
	Provided quickly or is available when needed	19	12 (63)	1 (5)	6 (32)	19 (100)
	Enables access to information previously unavailable or accessed through another process	9	4 (44)	2 (22)	3 (33)	9 (100)
	Overall quality of information	8	3 (38)	1 (13)	4 (50)	8 (100)
	Standards, coding, or documentation for data storage and retrieval	8	4 (50)	1 (13)	3 (38)	8 (100)
	Layout and format	6	3 (50)	0 (0)	3 (50)	6 (100)
	Subtotal	72	38 (53)	10 (14)	24 (33)	72 (100)
**User satisfaction**					
	Perceived usefulness or value and trust or confidence in system	22	17 (77)	2 (9)	3 (14)	22 (100)
	Integrated into workflow	19	8 (42)	5 (26)	6 (32)	19 (100)
	Ease of use	13	3 (23)	4 (31)	6 (46)	13 (100)
	Overall satisfaction	11	4 (36)	5 (45)	2 (18)	11 (100)
	Subtotal	65	32 (49)	16 (25)	17 (26)	65 (100)
**System quality**					
	Performance and reliability	22	6 (27)	3 (14)	13 (59)	22 (100)
	Security and privacy	16	6 (38)	1 (6)	9 (56)	16 (100)
	Assessment and planning	6	5 (83)	0 (0)	1 (17)	6 (100)
	Subtotal	44	17 (39)	4 (9)	23 (52)	44 (100)
**Service quality**					
	Training and support	18	9 (50)	4 (22)	5 (29)	18 (100)
	Stakeholder engagement	17	5 (29)	2 (12)	10 (59)	17 (100)
	Subtotal	35	14 (40)	6 (17)	15 (43)	35 (100)
Total		522	298 (57)	107 (20)	117 (22)	522 (100)

^a^iEHR: interoperable electronic health record.

^b^HIE: health information exchange.

When looking at the results from a setting-specific perspective, where sufficient volumes existed, there were some notable differences from the overall results. Acute care settings were assessed by 59 out of 113 studies (52.2%) and represented 270 out of 522 (51.7%) distinct measurement outcomes. Of these, there was focus on each of the six dimensions, with considerable attention on service quality, system quality, and some aspects of productivity. Primary care settings were assessed by 44 out of 113 (38.9%) studies and represented 183 out of 522 (35.1%) distinct measurement outcomes that covered most of the six dimensions, with attention directed predominantly to productivity measures while service, system, and information quality received less focus. Emergency department settings were assessed by 34 out of 133 (30.1%) studies and represented 112 out of 522 (21.5%) distinct measurement outcomes. There was a lack of measurement outcomes for most of the six dimensions, with the exception of one type of productivity item and one type of quality item.

### Summary of Findings From Reviews

We identified 17 reviews on iEHR use or impact (see Table 2). Of these reviews, 6 (35%) focused on barriers and facilitators to adoption or implementation, and 11 (65%) focused on benefits or impacts. A total of 10 of the 17 reviews (59%) were published between 2013 and 2016 and, with the exception of 3 reviews (18%) that were limited in scope to clinical research [13], chronic disease [14], or ambulatory primary care [15], most reviews (14/17, 82%) examined general benefits or impacts of iEHRs or HIEs.

The more recent reviews (ie, published since 2015) found little generalizable evidence of benefit or impact. The reviews highlight some less methodologically robust research that focused on resource use and perception of outcomes; these reviews found that iEHRs or HIEs increase productivity (eg, reduction in duplicate testing, emergency department costs, or hospital admissions [16,17]) and are valued by patient and physician stakeholders [17], all of which is consistent with the findings from our review. However, authors of one of the recent reviews [18] cautioned on overinterpreting the generalizability of this work given the methodological limitations of the primary studies and the developing state of iEHR or HIE evaluative work overall. The recent reviews on the benefit or impact of iEHRs or HIE identify three main areas for future research, including the following: (1) focus on how the setting in which iEHRs or HIEs are used affects specific health care outcomes [18]; (2) use of more rigorous, coordinated, and systematic approaches to evaluate the relationship between iEHRs or HIEs and health care outcomes [16]; and (3) need for better understanding of the organizational factors that affect iEHR or HIE contributions to improved clinical care [17].

**Table 2:** Summary of review articles included in this review.

Authors	Title	Source	Year	Primary focus
Anonymous [14]	Electronic tools for health information exchange: An evidence-based analysis	Ontario Health Technology Assessment Series. 13 (11).	2013	Benefits and impacts
Akhlaq et al [19]	Barriers and facilitators to health information exchange in low- and middle-income country settings: A systematic review	Health Policy & Planning. 31(9):1310-1325.	2016	Barriers and facilitators
Dobrev et al [20]	Report on methodology for evaluating the socio-economic impact of interoperable EHR and ePrescribing systems	EHR IMPACT. Prepared for the European Commission, Directorate General Information Society and Media, Brussels.	2008	Benefits and impacts
Eden et al [21]	Barriers and facilitators to exchanging health information: A systematic review	International Journal of Medical Informatics. 88:44-51.	2016	Barriers and facilitators
Edwards et al [22]	Barriers to cross-institutional health information exchange: A literature review	Journal of Healthcare Information Management. 24(3):22-34.	2010	Barriers and facilitators
Flott et al [23]	A patient-centered framework for evaluating digital maturity of health services: A systematic review	Journal of Medical Internet Research. 18(4):e75.	2016	Benefits and impacts
Fontaine et al [15]	Systematic review of health information exchange in primary care practices	Journal of the American Board of Family Medicine. 23(5):655-670.	2010	Benefits and impacts
Hersh et al [16]	Outcomes from health information exchange: Systematic review and future research needs	Journal of Medical Internet Research. 17(12):e39.	2015	Benefits and impacts
Hincapie and Warholak [24]	The impact of health information exchange on health outcomes	Applied Clinical Informatics. 2(4):499-507.	2011	Benefits and impacts
Johnson and Gadd [25]	Playing smallball: Approaches to evaluating pilot health information exchange systems	Journal of Biomedical Informatics. 40(6 Suppl):S21-S26.	2007	Barriers and facilitators
Joshi [26]	Clinical value-add for health information exchange (HIE)	Internet Journal of Medical Informatics. 6(1).	2010	Benefits and impacts
Kruse et al [27]	Barriers over time to full implementation of health information exchange in the United States	JMIR Medical Informatics. 2(2):e26.	2014	Barriers and facilitators
Mastebroek et al [28]	Health information exchange in general practice care for people with intellectual disabilities: A qualitative review of the literature	Research in Developmental Disabilities. 35(9):1978-1987.	2014	Barriers and facilitators
Parker et al [13]	Health information exchanges: Unfulfilled promise as a data source for clinical research	International Journal of Medical Informatics. 87:1-9.	2016	Benefits and impacts
Rahurkar et al [18]	Despite the spread of health information exchange, there is little evidence of its impact on cost, use, and quality of care	Health Affairs. 34(3):476-483.	2015	Benefits and impacts
Rudin et al [17]	Usage and effect of health information exchange: A systematic review	Annals of Internal Medicine. 161(11):803-811.	2014	Benefits and impacts
Vest and Jasperson [29]	What should we measure? Conceptualizing usage in health information exchange	Journal of the American Medical Informatics Association. 17(3):302-307.	2010	Benefits and impacts

## Discussion

### Principal Findings

Consideration of the review results against the benefits evaluation framework provides a lens to assess where evaluative work has been targeted and where there may be gaps where future evaluative efforts should focus. A total of 57.1% (298/522) of all measurement outcomes were positive. Quality of care (88/136, 64.7%) and productivity (109/170, 64.1%) were the dimensions with the highest percentage of positive measurement outcomes. Prominent themes in the quality of care category were around coordination of care and clinical decision support. For the productivity dimension, efficiency in clinical processes, time savings, and costs were the prominent themes. The left side of the benefits evaluation framework (see Figure 1), including system, service, and information quality, as well as user satisfaction, had relatively lower proportions of positive measurement outcomes ranging from 39%-53%. Many of the factors critical to achieving quality and productivity benefits require concentrated efforts on the left side of the framework. Change management efforts and other studies evaluating the benefits of iEHRs and other information systems suggest that user satisfaction increases when users have access to high-performing technology that is well integrated into their workflow, interoperable with existing systems, and is able to provide them with the information they need when they need it [2,12,30,31]. In addition, appropriate levels of support and training are necessary to ensure use of information systems.

Overall, our findings suggest that positive results tend to attract more evaluation, which may be explained by efforts to use progressively more rigorous methodologies, but also may reflect inefficient allocation of limited evaluation resources that could be applied to less-studied aspects of iEHRs and HIEs. The findings also point to several broader evaluation dimensions and several specific measurement items that require more attention going forward, including use and access, for which we did not identify any measurement items, and the service quality, system quality, user satisfaction, and information quality dimensions, which had fewer measurement items than productivity and quality dimensions. It is important to note that we did not perform quality appraisals of the primary studies; therefore, these review results need to be interpreted cautiously, which is a general theme of the reviews we assessed. While promising work exists, there is a clear need for more rigorous and comprehensive evaluation, with priority to support methodologies that can produce high-quality evidence. Overall, the review findings highlight the need to support more robust and comprehensive evaluative work across Canada on the impact of connected health information, covering more disease domains, health care settings, and populations.

### Limitations

This review identified a large number of studies that address the use and impact of connected health information through iEHRs and HIEs. The majority of the studies have been published within the last 5 years, which reflects a developing rather than mature evidence base. Given that the bulk of this evidence base is current, concerns regarding potential temporal biases that might not accurately reflect the quickly evolving developments of iEHRs and HIEs should be limited. However, our analysis did not assess whether systematic temporal differences in dimension-specific evaluations were present (eg, service and quality evaluated sooner after system launch vs productivity evaluated at more mature stages following launch). Consistent with the developing nature of the evidence base, it is notable that a sizable proportion of the articles identified in this review (12/130, 9.2%) came from grey literature sources, highlighting broader contributions to iEHR and HIE evaluation. Beyond the United States, which is the focus of the vast majority of the primary studies identified, we found few other primary studies globally, with Israel a distant second in terms of evaluative work on iEHRs and HIEs, followed by Canada, Finland, and the United Kingdom. Given fundamental differences in the organization of health systems and services and the dearth of evaluations in non-US settings, there are limits on how generalizable these assessments across jurisdictions will be.

### Conclusions

In conclusion, this review captures evaluative work from an evolving and active field focused on the use and impact of iEHRs and HIEs. While the overall findings suggest many positive impacts of iEHRs and HIEs, the quality of the primary studies were not evaluated systematically. When broken down by specific measurement items, some measurement outcomes consistently presented positive outcomes, while others were mostly negative or equivocal, highlighting areas for more attention. Setting-specific findings provide further insight on where more evaluative attention is needed.

## Appendix

Multimedia Appendix 1 Database-specific search strategies.

## Abbreviations

CINAHL: Cumulative Index to Nursing and Allied Health Literature
EHR: electronic health record
HIE: health information exchange
iEHR: interoperable electronic health record
PRESS: Peer Review of Electronic Search Strategies
PRISMA: Preferred Reporting Items for Systematic Reviews and Meta-Analyses
