# Supplementary material for: Interoperable Electronic Health Records and Health Information Exchanges: Systematic Review
Source: JMIR Med Inform. 2019 Jun 6;7(2):e12607. doi: 10.2196/12607 (PMC6592487; doi:10.2196/12607)
Supplement: Multimedia Appendix 1 [file medinform_v7i2e12607_app1.pdf]

Appendix 1: Database-Specific Search Strategies

MEDLINE (Ovid) January 25, 2017

#	Searches
1	(interoperab$ adj5 electronic health record).mp. [mp=title, abstract, original title, name of 53 Advanced substance word, subject heading word, keyword heading word, protocol supplementary concept word, rare disease supplementary concept word, unique identifier]
2	(interoperab$ adj5 ehr).mp. [mp=title, abstract, original title, name of substance word, subject 115 heading word, keyword heading word, protocol supplementary concept word, rare disease supplementary concept word, unique identifier]
3	(shar$ adj5 electronic health record).mp.
4	(shar$ adj5 ehr).mp.
5	(health information adj5 exchang$).mp.
6	1 or 2 or 3 or 4 or 5
7	limit 6 to yr="2006 -Current"
8	limit 7 to english language

EMBASE (Ovid) January 25, 2017

#	Searches
1	(interoperab$ adj5 electronic health record).mp. [mp=title, abstract, heading word, drug trade 49 Advanced name, original title, device manufacturer, drug manufacturer, device trade name, keyword, floating subheading]
2	(interoperab$ adj5 ehr).mp. [mp=title, abstract, heading word, drug trade name, original title, device manufacturer, drug manufacturer, device trade name, keyword, floating subheading]
3	(shar$ adj5 electronic health record).mp.
4	(shar$ adj5 ehr).mp.
5	(health information adj5 exchang$).mp.
6	1 or 2 or 3 or 4 or 5
7	limit 6 to yr="2006 -Current"
8	limit 7 to english language

CINAHL (EBSCOHost) January 25, 2017

#	Searches
7	S1 OR S2 OR S3 OR S4 OR S5<br>**Limiters** - Published Date: 20060101- 20171231; English Language
6	S1 OR S2 OR S3 OR S4 OR S5
5	(health information n5 exchang*)
4	(shar* n5 ehr)
3	(shar* n5 electronic health record)
2	(interoperab* n5 ehr)
1	(interoperab* n5 electronic health record)

google.ca February 9, 2017

#	Searches
1	"electronic health record" OR EHR AND interoperability AND evaluation
2	"health information exchange" OR HIE AND evaluation

scholar.google.ca February 9, 2017

#	Searches
1	"electronic health record" OR EHR AND interoperability AND evaluation
2	"health information exchange" OR HIE AND evaluation

greylit.org February 9, 2017

#	Searches
1	electronic health record AND interoperable [in title]
2	electronic health record AND shared [in title]
3	HIE [in title]
4	health information exchange [in title]

opengrey.eu February 9, 2017

#	Searches
1	"electronic health record"
2	"health information exchange" or HIE
3	EHR
